# A national Swedish case-control study investigating incidence and factors associated with idiopathic intracranial hypertension

**DOI:** 10.1177/03331024211024166

**Published:** 2021-08-18

**Authors:** Anna Sundholm, Sarah Burkill, Elisabet Waldenlind, Shahram Bahmanyar, A Ingela M Nilsson Remahl

**Affiliations:** 1 Department of Clinical Neuroscience, Karolinska Institutet, Stockholm, Sweden and Department of Neurology, 59562 Karolinska University Hospital, Karolinska University Hospital, Stockholm, Sweden; 2 Saw Swee Hock School of Public Health, 37580 National University of Singapore, National University of Singapore, Singapore; 3 Centre for Pharmacoepidemiology, Department of Medicine, Solna, Karolinska Institutet, Stockholm, Sweden and Centre for Phychiatry Research, Karolinska Institutet, Stockholm, Sweden

**Keywords:** Idiopathic intracranial hypertension, pseudotumor cerebri syndrome, risk factors, incidence, case-control study

## Abstract

**Objective:**

To study the incidence of idiopathic intracranial hypertension in Sweden and to explore whether previously proposed risk factors are associated with idiopathic intracranial hypertension by investigating the odds of exposure one year prior to diagnosis in patients compared to controls.

**Methods:**

Using Swedish health care registers and validated diagnostic algorithms, idiopathic intracranial hypertension patients diagnosed between 2000–2016 were compared with randomly selected matched controls, five from the general population and five with obesity.

**Results:**

We identified 902 idiopathic intracranial hypertension patients and 4510 matched individuals in each control group. Mean incidence among inhabitants ≥18 years of age was 0.71 per 100,000; rising from 0.53 in 2000–2005 to 0.95 in 2012–2016. There were increased odds for idiopathic intracranial hypertension patients compared to general population for exposure to: kidney failure (odds ratio =13.2 (4.1–42.0)), arterial hypertension (odds ratio =17.5 (10.5–29.3)), systemic lupus erythematosus (odds ratio =13.8 (4.3–44.7)), tetracyclines, sulphonamides, lithium, and corticosteroids. In obese controls, odds ratios were also significantly increased for these exposures. Hormonal contraceptive use and exposure to pregnancy did not appear to be associated factors for idiopathic intracranial hypertension development.

**Conclusions:**

The incidence of idiopathic intracranial hypertension in Sweden is lower relative to reports from other countries but is on the rise. This case-control study confirms several previously reported risk factors associated with idiopathic intracranial hypertension.

## Background

Idiopathic intracranial hypertension (IIH) is a condition resulting in high intracranial pressure (ICP) without known cause. The incidence of IIH among adults varies between countries from 0.03 to 4.7 per 100,000 inhabitants (1,2), with a reported pooled incidence of 1.2 per 100,000 (1). In Sweden incidence was reported to be 0.65 per 100,000 inhabitants (3).

Even though the pathophysiology remains unclear, several potential risk factors associated with the disorder have been described over the years. Female sex and obesity have the strongest association with IIH (4).

Other possible risk factors include Addison´s disease, hyperparathyroidism, systemic lupus erythematosus (SLE), kidney failure, iron deficiency anaemia, and exposure to tetracyclines, sulphonamides, cycline antibiotics, vitamin A, lithium, hormonal contraceptives and pregnancy (4). For some of these factors, the strength of the association has been questioned (4). There are few previous case-control studies available with a representative control population investigating risk factors associated with IIH, most including between 20–60 cases (5–9) aside from two studies investigating fluoroquinolones antibiotics (339 cases) (10) and hormonal contraceptives (3323 cases) (11). Otherwise knowledge on risk factors is based primarily on case reports or reviews (4,12). Therefore, further evaluations of risk factors are needed.

The aim of this national study was to investigate the incidence of IIH in Sweden and to study whether IIH patients are more frequently exposed to previously described factors associated with IIH in the year prior to IIH diagnosis compared to matched controls.

## Methods

### Study population

All inhabitants ≥18 years old in Sweden were included as the source population. Inclusion criteria were all patients with an incident IIH diagnosis code (using the International Classification of Diseases - tenth edition (ICD-10) code: G93.2) during the years 2000–2016 fulfilling predefined algorithms. The algorithms were developed to improve identification of true cases since the validity of IIH diagnostic codes is reported to be low (65%) (3). These algorithms, that were developed and tested in our previous validation study (diagnosis of IIH defined using the modified Dandy criteria), use the parameters of age and having the diagnosis code G93.2 recorded ≥3 times (13). A second algorithm also included acetazolamide treatment. If the predicted probability from the algorithm was ≥0.5, the algorithm was deemed to have predicted that the patient had true IIH. The algorithms improved the prediction of a correct diagnosis of IIH to 86–88% (13). Each IIH case was individually matched to five randomly selected individuals from the general population (GP) and five obese controls defined as those given a diagnosis code for obesity (E66). Data on weight or body mass index (BMI) were not available. Matching factors were age, sex, region of residence and vital status. Index date is the date of first diagnosis code of G93.2 for IIH cases and the same index date is used for each matched control.

For the study of exposure to drugs cases and controls with an index date between 1 July 2006 and 31st December 2016 were included.

### Register information

All registers used in the study have national coverage. The register data can be linked by using the unique personal identity number (PIN) that all inhabitants in Sweden are given at birth or immigration.

All specialized clinics and departments in Sweden are obliged to register all relevant diagnosis codes using ICD-10 to the Swedish National Patient Register (NPR) for every outpatient and inpatient visit. Data on possible risk factor diagnoses were obtained from this register from its ICD-10 coding (Table 1 includes codes used). The NPR comprises data on age, sex, date of admission and discharge, hospital, clinic, main and secondary diagnoses, for all contacts with specialized in- and out-patient visits. Primary care visits are not included in the NPR. Validation of diagnosis codes in the NPR has shown good validity of registered diagnoses with a PPV of between 85–95% depending on the disease (14).

**Table 1.** Frequency of registered diagnosis code for different previously suggested risk factor disorders and pregnancy exposure.

Registered diagnosis code one year prior to index date (ICD-10 code)	IIH N:902 n (%)	GP controls N: 4510 n (%)	Obese controls N:4510 n (%)
Addison (E27)	<4 (≤0.3)	<4 (<0.1)	<4 (<0.1)
Arterial hypertension (I10-15)	72 (8.0)	23 (0.5)	91 (2.0)
Coagulopathy (D65-68)	6 (0.7)	<4 (<0.1)	9 (0.2)
Cushing (E24)	<4 (≤0.3)	<4 (<0.1)	<4 (<0.1)
Hyperparathyroidism (E21)	<4 (≤0.3)	<4 (<0.1)	<4 (<0.1)
Hyperthyroidism (E05)	<4 (≤0.3)	8 (0.2)	12 (0.3)
Iron deficiency anaemia (D50)	9 (1.0)	5 (0.1)	10 (0.2)
Kidney failure (N17-19)	11 (1.2)	4 (0.1)	5 (0.1)
Ovary dysfunction incl PCOS in females (E28)	12 (1.6)	9 (0.2)	39 (1.0)
Pregnancy exposure in females (pregnancy register data)	56 (7)	262 (7)	272 (7)
Sarcoidosis (D86)	<4 (≤0.3)	<4 (<0.1)	7 (0.2)
SLE (M32)	11 (1.2)	4 (0.1)	6 (0.1)
Turner’s and Down’s syndrome (Q96+Q90)	<4 (≤0.3)	<4 (<0.1)	5 (0.1)
**Sensitivity analysis**			
Benign skin tumors (D22-23)	11 (1.2)	33 (0.7)	36 (0.8)

IIH = idiopathic intracranial hypertension, GP = general population, PCOS = polycystic ovary syndrome, SLE= systemic lupus erythematosus. Used ICD-10 codes in brackets.

The Prescribed Drug Register (PDR) was used to obtain data on drug dispensations using Anatomic Therapeutic Chemical (ATC) code information to identify type of drugs (Table 2 includes codes used). The PDR provides information on all pharmacological prescriptions and drug withdrawals from pharmacies in Sweden since 1st of July 2005, including from primary care, but does not include data on drug use during stays in hospitals.

**Table 2.** Frequency of exposure to previously suggested pharmacological risk factors for IIH.

Pharmacy dispensed medication the year prior to index date (ACT code)	IIH cases N = 654 n (%)	GP controls N = 3270 n (%)	Obese controls N = 3270 n (%)
Androgen treatments (G03B)	4 (0.6)	4 (0.1)	6 (0.2)
Contraceptives in women (G03A)	121 (22)	806 (29)	681 (25)
Lithium (N05AN)	10 (2)	6 (0.2)	11 (0.3)
Retinoidal derivatives for acne (D10BA)	<4 (<0.5)	4 (0.1)	<4 (<0.1)
Sulphonamides (J01EB-E)	9 (1)	<4 (<0.1)	12 (0.4)
Systemic corticosteroids (H02)	97 (15)	103 (3)	178 (5)
Tetracycline derivatives (J01A)	80 (12)	123 (4)	186 (6)
Quinolone derivatives (J01M)	22 (3)	39 (1)	64 (2)
**Sensitivity analysis**			
Antihypertensive treatments (C02-03, C07-09)	160 (24)	146 (4)	384 (12)
Iron deficiency anaemia treatments (B03A)	31 (5)	58 (2)	226 (7)

IIH  =  idiopathic intracranial hypertension, GP  =  general population. Used ACT codes in brackets.

Statistics Sweden (SS) provides information on living area, migration, deaths, and educational level and was used to select matched controls.

The Swedish Medical Birth Register (MBR) was used to identify exposure to pregnancy. The MBR provided data on pregnancy length and date of delivery (year + month) if the pregnancy lasted 22 weeks or longer (15). Data on spontaneous and elective abortions are not available.

### Design, outcome and exposures

We firstly evaluated the incidence and change of the incidence over time of the included IIH cases after use of algorithms.

Secondly, we assessed whether exposures identified as risk factors in previously published studies were associated with IIH using our case-control data (Tables 1 and 2). Cases and controls were regarded as exposed if there was at least one record during the year preceding the index date in the registers for the risk factor being studied. Pregnancy exposure was defined as at least a three-month exposure ± 15 days (of being pregnant) during the year prior to index date. As a sensitivity analysis we investigated benign skin tumours since we did not expect benign skin tumours to be influenced by obesity, inflammatory activation, hormonal imbalance or to be associated with IIH. As for arterial hypertension and iron deficiency anaemia we also conducted a sensitivity analysis by evaluating exposure to drugs used to treat these disorders. This was conducted as these disorders are mostly treated and followed within primary care, for which data is lacking in the NPR.

### Statistics

The incidence was obtained by recording how many new cases of IIH (after use of algorithms) were recorded each year divided by the total source population ≥18 years old for each year (sum of living individuals at the end of each year (31st December)).

We used conditional logistic regression to estimate odds ratios (OR) and 95% confidence intervals (CI) for exposure to possible risk factors comparing IIH to GP controls as well as comparing IIH to obese controls. Odds ratios were not calculated for those disorders or treatments where we found less than four registered cases with included codes during the year prior to index date. In OR calculations we adjusted for educational level (categorized as level 1: ≤ 9 years of compulsory school, level 2: secondary school, level 3: higher education, collage/university). STATA 12 was used to undertake the statistical analyses.

### Ethics

The study has approved ethical consent by the Regional Ethical Committee in Stockholm (2017/1291-31). Each register also accepted withdrawal of data in accordance with the ethical application. Informed consent is not required for use of de-identified register data.

## Results

During the study period 2000–2016 in total 1439 persons had a first-time diagnosis code of G93.2. After applying the algorithms, 902 individuals (63%) were predicted to be correctly diagnosed with IIH. Five individually matched controls gave 4510 general population (GP) controls and 4510 obese controls. The cases and controls had a mean age of 32 with a female/male sex ratio of 5.7:1 (Table 3). Educational level differed among cases and controls (Table 3) and was used in the adjusted model as a proxy for socioeconomic status. When assessing drug dispensation (July 2006–2016) we included 654 IIH cases, 3270 GP controls, and 3270 obese controls.

**Table 3.** Demographics.

	IIH	GP controls	Obese controls
Participants n	902	4510	4510
Age at diagnosis/index date (years): mean (SE)	32.2 (0.17)	32.2 (0.17)	32.2 (0.17)
median (IQR)	29 (23–38)	29 (23–38)	29 (23–38)
Information on achieved highest educational level n (%)	887 (98.3%)	4454 (98.8%)	4453 (98.7%)
Compulsory school (≤9 years)	163 (18.4%)	439 (9.9%)	766 (17.2%)
Upper secondary (high school)	447 (50.4%)	1853 (41.6%)	2408 (54.1%)
University (level above high school)	277 (31.2%)	2162 (48.5%)	1279 (28.7%)

GP = General population, n=number, SE = Standard Error, IQR= interquartile range.

### Incidence of IIH in Sweden

**Figure 1.**
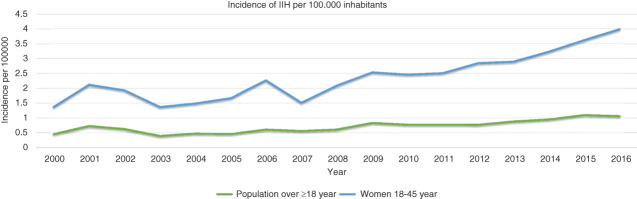
Incidence of IIH in Sweden 2000–2016.

Over the study period the mean yearly incidence in Sweden for those ≥18 years of age was 0.71 per 100,000 inhabitants overall; among females aged 18–45 the incidence was 2.35 per 100,000, and in males aged 18–45 the incidence was 0.32 per 100,000. There were increasing incidences over the years in both sexes and in all age groups (see Table 4). The increase was most pronounced among females age 18–45 in whom the incidence doubled between the first six years compared to the last five years of the study period (Table 4 and Figure 1).

**Table 4.** Mean yearly incidence of IIH per 100,000 inhabitants in Sweden 2000-2016 by investigated time periods, age range and sex.

Sex	Age range (years)	Mean yearly incidence / time-period:			Mean yearly incidence:
		2000–2005	2006–2011	2012–2016	2000–2016
Male + Female	≥18	**0.53**	**0.70**	**0.95**	**0.71**
	≥18 to <30	1.47	1.92	2.53	**1.94**
	≥30 to <40	0.61	1.15	1.37	**1.02**
	≥40 to <50	0.43	0.42	0.84	**0.55**
	≥50	0.16	0.14	0.20	**0.16**
Female	≥18 to ≤45	1.66	2.23	3.32	**2.35**
Male	≥18 to ≤45	0.20	0.41	0.37	**0.32**

### Previously proposed factors associated with IIH

**Figure 2.**
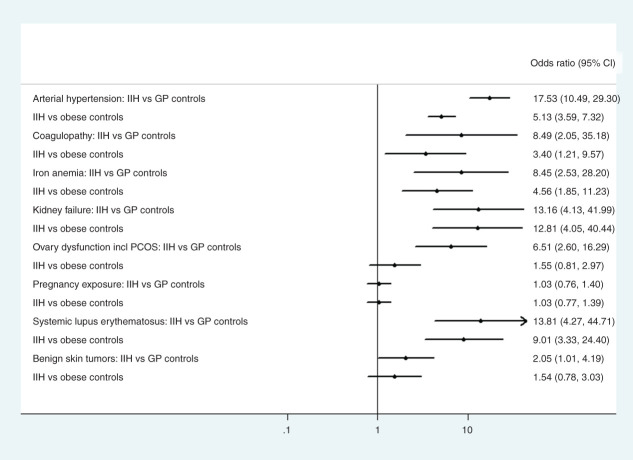
Forest plot comparing exposure to previously reported risk factors, diagnoses and pregnancy, in IIH patients compared to controls.

The odds were significantly increased compared to both control groups for the following disorders: kidney failure, iron deficiency anaemia, arterial hypertension, systemic lupus erythematosus (SLE), coagulopathy, (OR between 8.4–17.5 compared to GP controls and 3.4–12.8 compared to obese controls), see Figure 2, Supplementary Table 1. The odds ratio for having ovarian dysfunction including polycystic ovary syndrome (PCOS) in females was significantly increased compared to GP controls, OR=6.5 (95% CI 2-6-16.3) but not compared to obese controls, OR 1.6 (95% CI 0.8–3.0). Sarcoidosis, Turner´s syndrome, Down´s syndrome, Addison, Cushing´s disease, hyperthyroidism and hyperparathyroidism were too rare (n<4) to allow for informative estimates of whether IIH risk was increased or not, (see frequency Table 1). When analysing pregnancy exposure there was no difference between IIH patients and either of the control groups.

As a sensitivity analysis we investigated benign skin tumours. This sub-analysis did show a borderline to no significant difference between cases and controls affected by this disorder (Figure 2, Supplementary Table 1).

**Figure 3.**
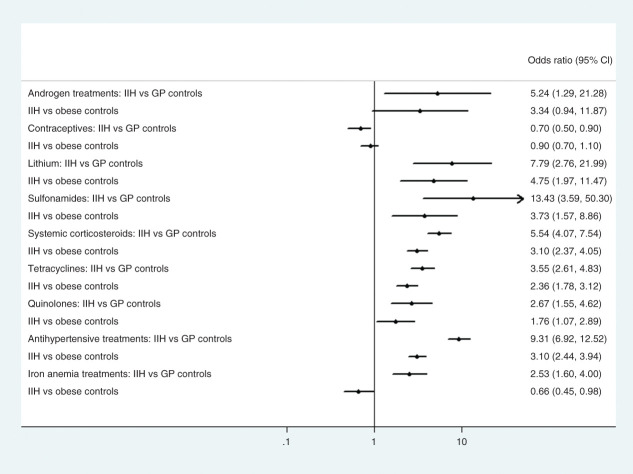
Forest plot comparing exposure to previously reported risk factor drugs in IIH patients compared to controls.

Among reported pharmacological factors associated with IIH, those exposed to tetracyclines, sulphonamides, quinolones, corticosteroids, and lithium all had significantly higher odds when comparing IIH patients to controls (OR between 2.7–13.4 compared to GP controls and OR 1.8–4.8 compared to obese), see Figure 3, Supplementary Table 2. Hormonal contraceptives were less frequently used in IIH cases compared to both control groups. Exposure to retinoidal treatments were too low to conduct a reliable comparison (see frequency Table 2). The OR for androgen treatments exposure were only significant relative to the GP controls but the numbers were low. Sensitivity analysis on antihypertensive drug dispensation confirmed the high ORs obtained for arterial hypertensive diagnosis. However, sensitivity analysis on iron deficiency anaemia treatments resulted in significantly increased OR when comparing IIH to GP controls but not compared to obese controls (see Figure 3, Supplementary Table 2).

## Discussion

### Incidence

In the general population over 18 years of age the mean incidence of IIH in the Swedish population was in the lower range of reported incidences, being 0.95 per 100,000 inhabitants per year during the latter part of the study period, 2012–2016, compared to 1.2 per 100,000 in the meta-analysis from 2018 (1). Our results from this national based study are consistent with the results from our previous study of incidence in one county of Sweden (3).

This study also confirms previous reports on an increasing incidence of IIH (2,16–18) however the incidences in these countries are higher compared to what is reported in this study. A much higher incidence of IIH is also reported in a recent study from Kuwait (19). These other countries, however, report a much higher obesity prevalence compared to reported prevalence from Sweden (20). The incidence in this study increased by 79% when comparing the first 6 years with the last 5 in the investigated period. Increase in obesity prevalence during this time period is reported in Sweden (20). This is probably the main explanation to the seen differences in incidence of IIH between countries and over time. However, we cannot exclude the possibility differences in results are due to different methodologies, particularly since previous validation studies of IIH diagnosis coding, including our own, have shown that an incorrect diagnosis is common (35–40% incorrect or later changed diagnosis) (3,21). This might have had an impact on part of the incidence difference in our study compared to the other register studies (2,18). We cannot exclude also a slight underestimation of the Swedish incidence due to our algorithm predicting patients to be less likely to be true IIH patients if they receive two or less diagnosis codes of G93. To conclude, we believe that the rise in IIH incidence primarily may be explained by the increase in obesity seen worldwide, as well as in Sweden.

In this study the female: male ratio was 5.7 to 1. This was consistent with the results from our previous validation study (3) and in several incidence studies (1,2). However, larger sex differences, with a female: male ratio of 38:1 have also been reported (22). Why the sex difference varies to this large extent is unknown. We also found that highest educational level was much lower in IIH patients compared to GP controls. It has been reported that IIH seems to affect socioeconomic groups differently with higher risk of IIH in socially deprived groups (2,18,23), possibly due to inequalities in healthcare that should be highlighted.

### Factors associated with IIH

We have confirmed significantly increased odds for exposure to several of the previously reported conditions/pharmacological factors associated with IIH. Exposure to a diagnosis code of arterial hypertension surprisingly showed the strongest association, (OR 17.5) in IIH compared to GP controls but was also significantly elevated (OR 5.1) compared to obese controls. Sensitivity analyses on drug dispensations for anti-hypertensive drugs confirmed these results. The association between arterial hypertension and IIH could be explained by comorbidities (obesity being a risk factor for both disorders) but the increased OR compared to both control groups supports the argument that it might be a separate risk factor for IIH. The diagnosis arterial hypertension has been reported previously with a possible association with IIH in two small-scale case-control studies (6,8). Arterial hypertension is also shown to be particularly common amongst older IIH patients (>40 years, mean age 51) (24) and IIH patients have been shown to have a two-fold increased risk of developing cardiovascular disorders relative to matched controls (25). However, we cannot exclude the possibility that some cases may have had a hypertensive crisis (hypertensive encephalopathy) with papilledema and a similar clinical picture as IIH, or that measurement errors occurred due to not using a correct cuff in obese patients which could have resulted in over-diagnosis. There is some experimental evidence of a correlation between raised ICP and subsequent increased blood pressure due to sympathetic activity, and suggestions that keeping the intracranial pressure stable involves changes on either the arterial side (blood pressure) (26) or venous side (venous stenosis) (27). The association between IIH and intracranial venous stenosis successfully treated with stents have been described in several studies (28). It has also been proposed that venous stenosis disappears after normalization of intracranial pressure (29). Whether arterial hypertension is a primary risk factor or a secondary consequence of increased ICP is an important research question for further studies.

In the present study, exposure to kidney failure (as a proxy for uraemia) was much more common prior to IIH diagnosis compared to controls and a probable risk factor for IIH development (OR 13 compared to both control groups). The underlying pathophysiological cause of why uraemia is associated with IIH is unknown. End stage kidney failure with uraemia is associated with inflammation in the adipose tissue regardless of adiposity level (30) and we have previously proposed that possibly inflammatory activation could be involved as an underlying mechanism (31). Kidney failure is also associated with arterial hypertension which might be part of the pathophysiological process. SLE also turned out to be significantly more common prior to IIH development. SLE is classified as an inflammatory disorder and inflammation could again be a mechanism behind how this disorder is associated with IIH. SLE is also associated with increased risk for thrombosis, therefore we cannot rule out having included secondary cases of IH due to sinus thrombosis; which also applies to coagulation disorders that were also more prevalent in IIH compared to controls.

#### Pharmacological treatments

Our results support a possible treatment-associated risk, especially for tetracyclines, lithium and corticosteroids. This supports the results of a recent review of drug-induced intracranial hypertension (DIIH) (12) concluding that the evidence is highest for vitamin A derivatives, tetracyclines and lithium, and moderately associated with corticosteroidal withdrawals but not associated with other suggested antibiotics and hormonal contraceptives.

A recent study looking at cycline antibiotics reported an initial increased risk for development of IIH suggesting that the substance could be the risk factor, but when confounders were adjusted for this effect disappeared, and there was no dose-response effect that could strengthen the evidence base (32). When discussing pharmacological treatments as risk factors one must consider the indication why they are used. For antibiotics, the underlying infections might explain the increased risk seen as proposed in our previous study (31). In that study we found an increased OR on the proxy analysis of drug dispensation of antibiotics, even when the risk factor antibiotics (tetracyclines, sulphonamides, quinolones) were excluded. Still, the OR of exposure prior to IIH development in this study was slightly higher for tetracycline and sulphonamide antibiotics compared to all other antibiotics in the previous analysis (31), which supports the argument that there may be an effect from the drug itself. We also found increased OR for lithium and corticosteroid treatments. Lithium, commonly used to treat bipolar disorder, is proposed to affect inflammation in the CNS (33). The same applies to corticosteroid treatments as this treatment is often used to treat inflammatory disorders. Additionally, longstanding treatments with corticosteroids can cause weight gain, another strong risk factor for IIH. These proposed hypotheses require further research.

#### Non-conclusive or negative findings among previously suspected factors associated with IIH

We found no association with hormonal contraceptive treatments, rather the opposite, i.e. the exposure to hormonal contraceptives was lower in IIH than in GP controls. This supports previous reports that use of contraceptives containing oestrogen is not a risk factor for IIH development (4,6,8) and is in line with the results from Kilgore et al. (9). However, we cannot rule out that there might be an increased risk with certain types of hormonal contraceptives (11). Pregnancy exposure also did not increase the risk of IIH, as has been suggested previously (8,34).

A diagnosis of iron deficiency anaemia was more common in IIH patients relative to controls. When examining treatment dispensation for iron deficiency anaemia, this was only significant compared to GP controls, while lower OR were seen compared to obese controls. With these somewhat divergent results the question remains a matter of debate.

### Strengths and limitations

One strength of this study is that it has a national population-based design including all IIH cases in Sweden for 16 years, thereby capturing a decent sized IIH cohort. Another strength is the use of pre-determined algorithms which improves the prediction of “true cases” since several studies have shown that IIH is commonly misdiagnosed (3,21). Since IIH is almost exclusively diagnosed in the secondary care setting, almost all patients will be captured using the NPR. Another strength is that the coverage of the registers in Sweden is very good; NPR is reported to have ≤ 1% missing data for main diagnosis code at each visit (14).

A clear limitation to this study is the lack of primary care visits which are not included in the NPR, resulting in missing data on exposures for conditions generally treated in primary care practice (especially diagnoses such as systemic hypertension and iron deficiency anaemia). We compensate for this by studying drug dispensation records in the PDR and conducting sensitivity analyses on dispensation of drugs used to treat these disorders. Another limitation is that the developed algorithms were shown to be less sensitive in selecting out secondary IH which means that some cases included as IIH patients might have secondary intracranial hypertension (13). A further limitation is the risk of surveillance bias, because there may be a considerable delay between symptom onset and correct diagnosis. Tests for multiple potential diseases may then be conducted in specialized care leading to diagnosis of concomitant disorders coded in the NPR before IIH finally is recognized.

BMI is not available in the Swedish health registers. The obese controls were therefore an important group to include as obesity is a strong risk factor and might also be a confounder for several other risk factors. Our results describe the Swedish population; but similar results could be expected in settings with a comparable range of diseases and living conditions.

## Conclusion

Our study confirms that the incidence of IIH is rising in Sweden, with the largest increase in females of reproductive age. Increasing obesity prevalence is likely to be an important factor explaining this. This case-control study confirms an association with several other factors previously reported to be associated with IIH. We speculate how these factors might be associated with IIH development and suggest that pressure regulatory mechanisms may be involved (maybe arterial hypertension and development of sinus transversus stenosis are explanatory components). Also, inflammatory activation could be involved as speculated in our previous paper (31) which might partly explain why we see an association with some previously proposed risk factors (factors which also cause inflammation or are used to treat inflammatory disorders). This study only gives rise to hypotheses, but we recommend that the investigative work by clinicians and follow-up of IIH patients should include these factors for consideration and treatment to obtain the best clinical outcomes, as suggested in European Guidelines on IIH (35).

## Article Highlights

- Incidence of IIH in Sweden was 0.71 per 100,000 inhabitants which is in the lower range of IIH incidence reports in the adult population. The incidence increased over the investigated period as reported in other studies.
- This study provides confirmation of an association between IIH and the factors kidney failure, arterial hypertension and SLE.
- Exposure to previously reported risk factor drugs: tetracyclines, sulphonamides, lithium, and corticosteroids were more common among IIH patients compared to controls the year prior to first diagnosis of IIH.
- Exposure to pregnancy and hormonal contraceptives did not appear to be factors associated with IIH.

## Supplemental Material

sj-pdf-1-cep-10.1177_03331024211024166 - Supplemental material for A national Swedish case-control study investigating incidence and factors associated with idiopathic intracranial hypertension Click here for additional data file. Supplemental material, sj-pdf-1-cep-10.1177_03331024211024166 for A national Swedish case-control study investigating incidence and factors associated with idiopathic intracranial hypertension by Anna Sundholm, Sarah Burkill, Elisabet Waldenlind, Shahram Bahmanyar and A Ingela M Nilsson Remahl in Cephalalgia
