# Supplementary material for: A national Swedish case-control study investigating incidence and factors associated with idiopathic intracranial hypertension
Source: Cephalalgia. 2021 Aug 18;41(14):1427–36. doi: 10.1177/03331024211024166 (PMC8619724; doi:10.1177/03331024211024166)
Supplement: sj-pdf-1-cep-10.1177_03331024211024166 - Supplemental material for A national Swedish case-control study investigating incidence and factors associated with idiopathic intracranial hypertension [file sj-pdf-1-cep-10.1177_03331024211024166.pdf]

Supplementary table 1: Odds ratio of registered diagnosis code in IIH patients compared to controls.

**Type of disorder:**	**Comparison between**	**OR_{crude} (95% CI)**	**$OR_{adjusted}$* (95%CI)**
**Arterial hypertension**	**IIH vs GP controls**	**18.4 (11.1-30.5)**	**17.5 (10.5-29.3)**
	IIH vs obese controls	5.1 (3.6-7.3)	5.1 (3.6-7.3)
**Coagulopathy**	**IIH vs GP controls**	**10.0 (2.5-40.0)**	**8.5 (2.0-35.2)**
	IIH vs obese controls	3.3 (1.2-9.4)	3.4 (1.2-9.6)
**Iron deficiency anemia**	**IIH vs GP controls**	**10.6 (3.3-34.7)**	**8.4 (2.5-28.2)**
	IIH vs obese controls	4.5 (1.8-11.1)	4.6 (1.8-11.2)
**Kidney failure**	**IIH vs GP controls**	**13.8 (4.4-43.2)**	**13.2 (4.1-42.0)**
	IIH vs obese controls	13.1 (4.2-43.3)	12.8 (4.1-40.4)
**Ovary dysfunction incl PCOS**	**IIH vs GP controls**	**7.2 (2.9-17.7)**	**6.5 (2.6-16.3)**
	IIH vs obese controls	1.5 (0.8-2.9)	1.6 (0.8-3.0)
**Pregnancy exposure**	**IIH vs GP controls**	**1.1 (0.8-1.5)**	**1.0 (0.8-1.4)**
	IIH vs obese controls	1.0 (0.8-1.4)	1.0 (0.8-1.4)
**SLE**	**IIH vs GP controls**	**13.8 (4.4-43.2)**	**13.8 (4.3-44.7)**
	IIH vs obese controls	9.2 (3.4-24.8)	9.0 (3.3-24.4)
**Sensitivity analysis**			
**Benign skin tumors**	**IIH vs GP controls**	**1.7 (0.8-3.4)**	**2.1 (1.0-4.2)**
	IIH vs obese controls	1.5 (0.8-3.0)	1.5 (0.8-3.0)

IIH = idiopathic intracranial hypertension, GP = general population, SLE= systemic lupus erythematosus, PCOS = polycystic ovary syndrome. Analysis of Addison, Cushing, Turner and Downs syndrome, hyperthyroidism, hyperparathyroidism was not possible to perform due to few exposures among IIH cases.

Supplementary table 2: Odds ratios of pharmacological exposure in IIH patients compared to controls

**Type of medication**	**Comparison between**	**OR_{crude} (95% CI)**	**$OR_{adjusted}$ (95% CI)**
**Androgen treatments**	**IIH vs GP controls**	**5.0 (1.3-20.0)**	**5.2 (1.3-21.3)**
	IIH vs obese controls	3.3 (0.9-11.8)	3.3 (0.9-11.9)
**Contraceptives in women**	**IIH vs GP controls**	**0.7 (0.5-0.8)**	**0.7 (0.5-0.9)**
	IIH vs obese controls	0.8 (0.7-1.1)	0.8 (0.7-1.1)
**Lithium**	**IIH vs GP controls**	**8.3 (3.0-22.9)**	**7.8 (2.8-22.0)**
	IIH vs obese controls	4.8 (2.0-11.5)	4.8 (2.0-11.5)
**Sulfonamides**	**IIH vs GP controls**	**15.0 (4.1-55.4)**	**13.4 (3.6-50.3)**
	IIH vs obese controls	3.8 (1.6-8.9)	3.7 (1.6-8.9)
**Systemic corticosteroids**	**IIH vs GP controls**	**5.5 (4.1-7.5)**	**5.5 (4.1-7.5)**
	IIH vs obese controls	3.1 (2.4-4.0)	3.1 (2.4-4.1)
**Tetracyline derivatives**	**IIH vs GP controls**	**3.6 (2.7-4.9)**	**3.6 (2.6-4.8)**
	IIH vs obese controls	2.3 (1.8-3.1)	2.4 (1.8-3.1)
**Quinolone derivatives**	**IIH vs GP controls**	**2.9 (1.7-4.9)**	**2.7 (1.5-4.6)**
	IIH vs obese controls	1.8 (1.1-2.9)	1.8 (1.1-2.9)

**Sensitivity analysis**			
**Antihypertensive treatments**	**IIH vs GP controls**	**9.5 (7.1-12.7)**	**9.3 (6.9-12.5)**
	IIH vs obese controls	3.1 (2.4-3.9)	3.1 (2.4-3.9)
**Iron deficiency anemia treatments**	**IIH vs GP controls**	**2.8 (1.8-4.3)**	**2.5 (1.6-4.0)**
	IIH vs obese controls	0.7 (0.4-1.0)	0.7 (0.4-1.0)

IIH = idiopathic intracranial hypertension, GP = general population. Analysis of retinoidal treatments was not possible to perform due to few exposures in IIH cases.
